# Early calcium replacement after minimal blood transfusion is associated with adverse outcomes

**DOI:** 10.1007/s00068-026-03282-6

**Published:** 2026-08-07

**Authors:** W. Preston Hewgley, Russell L. Griffin, Emily Baird, Zain G. Hashmi, Samantha J. Baker, Jan O. Jansen, Jeffrey Kerby, John B. Holcomb, Rondi B. Gelbard

**Affiliations:** Department of Trauma and Acute Care Surgery, University of Alabama at Birmingham, 1808 7th Ave S., 615 Boshell Diabetes Bldg D673B, Birmingham, AL 35233 USA

**Keywords:** Transfusion-related hypocalcemia, Empiric calcium, Early calcium

## Abstract

**Purpose:**

Calcium disturbances are common in patients with traumatic injuries, exacerbated by shock and blood product resuscitation. Recently, empiric calcium supplementation has been recommended with the first unit of blood transfusion, despite a lack of data supporting this practice. We hypothesize that there are risks associated with calcium administration in patients receiving small volume transfusion.

**Methods:**

This is a retrospective review of patients admitted to our Level 1 trauma center from 2018 to 2024 who received 1–3 units of red blood cells (RBCs). Patients were stratified by calcium levels and whether they received early calcium supplementation. A logistic regression was used to estimate odds ratios for the association between calcium levels, calcium supplementation, and outcomes. The primary outcome was in-hospital mortality. Secondary outcomes included acute kidney injury, myocardial infarction, acute respiratory distress syndrome, and venous thromboembolism. The model was adjusted for age, sex, ISS and shock index.

**Results:**

A total of 2,680 patients were included. When stratified by calcium levels and supplementation status, patients were similar in terms of age, sex, mechanism and injury severity score, but shock index was higher among patients receiving calcium supplementation. After adjustment, hypocalcemic patients who received calcium supplementation had higher odds of developing acute kidney injury, acute respiratory distress syndrome, deep vein thrombosis, and pulmonary embolism (all *p* < 0.05).

**Conclusion:**

In trauma patients who received 1–3 units of RBCs, early calcium supplementation was associated with adverse outcomes. The use of early, empiric calcium supplementation in conjunction with minimal transfusion should be carefully considered.

**Supplementary Information:**

The online version contains supplementary material available at 10.1007/s00068-026-03282-6.

## Introduction

Calcium disturbances are common in trauma. A traditional viewpoint attributes trauma-associated hypocalcemia to citrate chelation from blood product transfusion, but calcium is also rapidly depleted by a variety of other mechanisms in trauma, including hepatic dysfunction, hypothermia, acute hemorrhage, and reduced citrate metabolism [1–4]. There is a degree of uncertainty whether these mechanisms are purely pathologic or if some may be a physiologic response to trauma [4]. Nonetheless, there is a clear association between severe hypocalcemia (< 1.0 mmol/L), ongoing transfusion and mortality [5, 6].

Early, empiric calcium supplementation has been advocated during transfusion for hemorrhagic shock to avoid hypocalcemia. Recognized early in the conception of damage control resuscitation, the adoption of empiric calcium supplementation in military trauma reflects both the physiologic need to counteract citrate toxicity and the practical limitations of real-time ionized calcium measurement in the austere setting [7]. The JTS Clinical Practice Guideline (CPG) advises giving 1 gram of calcium chloride (or 3 g of calcium gluconate) before, during or after the first unit of blood and then again after every four units, to maintain an ionized calcium level > 1-1.2 mmol/L [8]. The military experience has influenced civilian massive transfusion protocols (MTP), and the recent AAST/ACS-COT protocol for damage control resuscitation echoes the JTS’s recommendations [9]. Some EMS agencies have even expanded this approach to the prehospital environment, infusing calcium before the first unit of blood is transfused [10].

Although several non-randomized studies suggest a mortality benefit from early calcium administration among patients receiving massive transfusion [3, 11–13], there is a lack of evidence for benefit of routine calcium replacement after small volume transfusions. Calcium may be administered upon activation of MTP, but prior data show that up to 45% of patients meeting clinical triggers for MTP receive 3 units or less of RBCs [14]. We hypothesize that there are risks associated with calcium supplementation in patients receiving small volume transfusion when it is not clearly indicated. This study aims to evaluate outcomes of early calcium supplementation in trauma patients with minimal transfusion requirements.

## Methods

### Study design and patient population

This is a retrospective cohort study designed to evaluate the effect of early calcium supplementation in minimally transfused patients admitted to our Level 1 trauma center between January 2018 and December 2024. Inclusion criteria were age ≥ 18 years and administration of 1–3 units of red blood cells (RBCs) in the first 24 h. Patients were excluded if no calcium levels were recorded or if they were hospitalized for fewer than four days, since this is the typical timeframe to develop the outcomes of interest. Patients were stratified by calcium levels and supplementation status. This study was performed under Institutional Review Board exemption given its retrospective nature and use of de-identified data.

### Calcium administration

At our institution, there is no clinical protocol directing calcium supplementation, but in general, providers administer empiric calcium if they expect the patient to require multiple transfusions. Otherwise, calcium supplementation is typically goal-directed to correct serum calcium or ionized calcium to reference range (> 1.12 mmol/L). Patients were considered to receive early calcium supplementation if they received calcium gluconate or calcium carbonate within 6 h of injury. Because of the retrospective nature of this study, we were unable to determine whether the decision to administer calcium was empiric or goal-directed.

Additionally, data regarding pre-hospital blood and calcium administration was not available, and delivery of these therapies was at the discretion of the EMS providers, who often administer calcium empirically when patients receive prehospital blood transfusion.

### Study variables and outcomes

Demographics, injury characteristics (i.e., mechanism, Injury Severity Score [ISS]), physiologic parameters (i.e., pulse, systolic blood pressure, shock index [SI]), RBC units transfused, and calcium levels were collected from the blood bank records, trauma registry, and electronic medical records. Patients were considered hypocalcemic if the lowest calcium level measured in the emergency department was less than 8.4 mg/dL or lowest ionized calcium level less than 1.12 mMol/L based on our institutional laboratory ranges.

The primary outcome was in-hospital mortality. Secondary outcomes included acute kidney injury (AKI), myocardial infarction (MI), acute respiratory distress syndrome (ARDS), and venous thromboembolic events (VTE), the latter of which included deep vein thrombosis (DVT) and pulmonary embolism (PE). Additionally, infectious complications including superficial, deep and organ/space surgical site infection; severe sepsis; central line-associated bloodstream infection (CLABSI); and ventilator associated pneumonia (VAP) were combined into one infection outcome. Outcomes were measured throughout the patients’ index hospitalization and were defined from trauma registry data according to the National Trauma Data Standard™ (NTDS) 2021 Data Dictionary.

### Statistical analysis

Patient demographic, injury, and clinical characteristics were compared using a Fisher’s exact test or analysis of variance test for categorical and continuous variables, respectively; in addition, within-group comparisons were made between calcium supplementation status using a Fisher’s exact and t-test for categorical and continuous variables, respectively.

A logistic regression utilizing Firth’s penalized likelihood (due to the low number of events among the NormoCa/SUP group) was used to estimate odds ratios (ORs) and associated 95% confidence intervals (CIs) for the association between calcium levels and supplementation status and select clinical outcomes. Models were adjusted for age, sex, ISS, and SI on admission to the emergency department. For each outcome, measures of interaction effect including the attributable proportion (AP)—which estimates the proportion of the combined effect that is due to the interaction—and the interaction contrast ratio (ICD)—which measures the relative risk increase due to the interaction—were calculated. In a post-hoc analysis, models were stratified by the propensity of calcium supplementation, presenting the associations in forest plots; the propensity model of calcium supplementation included the predictors age; sex; injury mechanism (i.e., blunt or penetrating); shock index > 1; number of RBCs transfused; lactic acid level; hemoglobin level; hypocalcemia; ISS; and AIS ≥ 3 injury to the head, thorax, abdomen and pelvis, upper extremity, and lower extremity. The propensity score was categorized, based on sample sizes to ensure adequate maximum likelihood estimate model convergence, to 0–9%, 10–14%, 15–19%, and ≥ 20%.

## Results

**Fig. 1:**
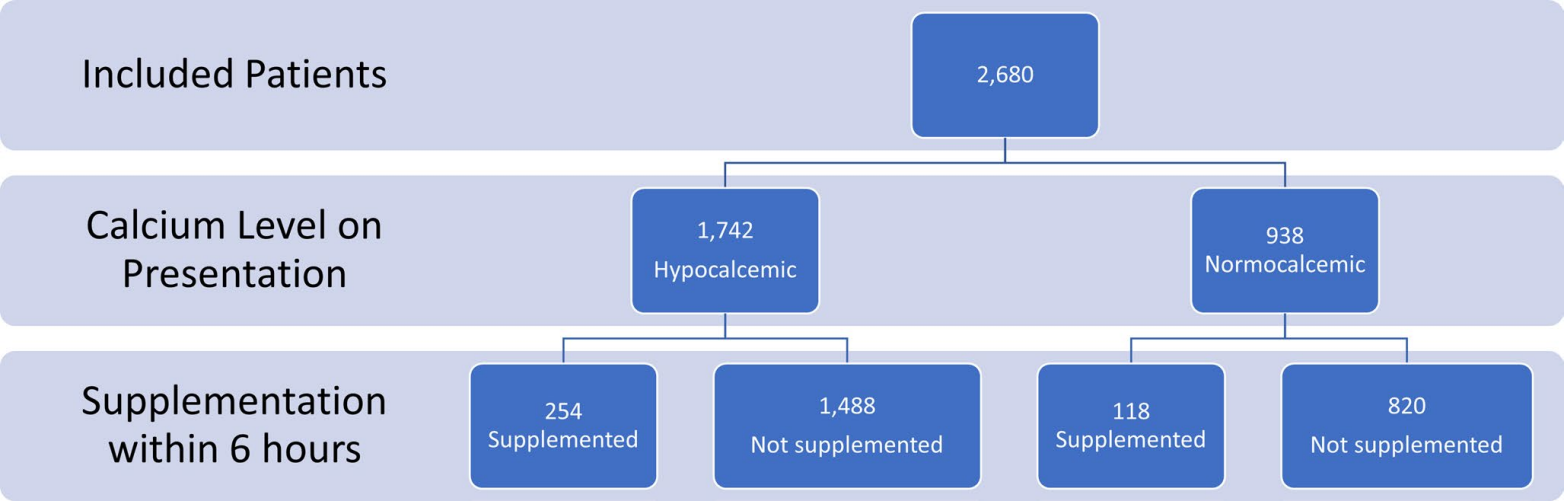
Patient population

**Fig. 2:**
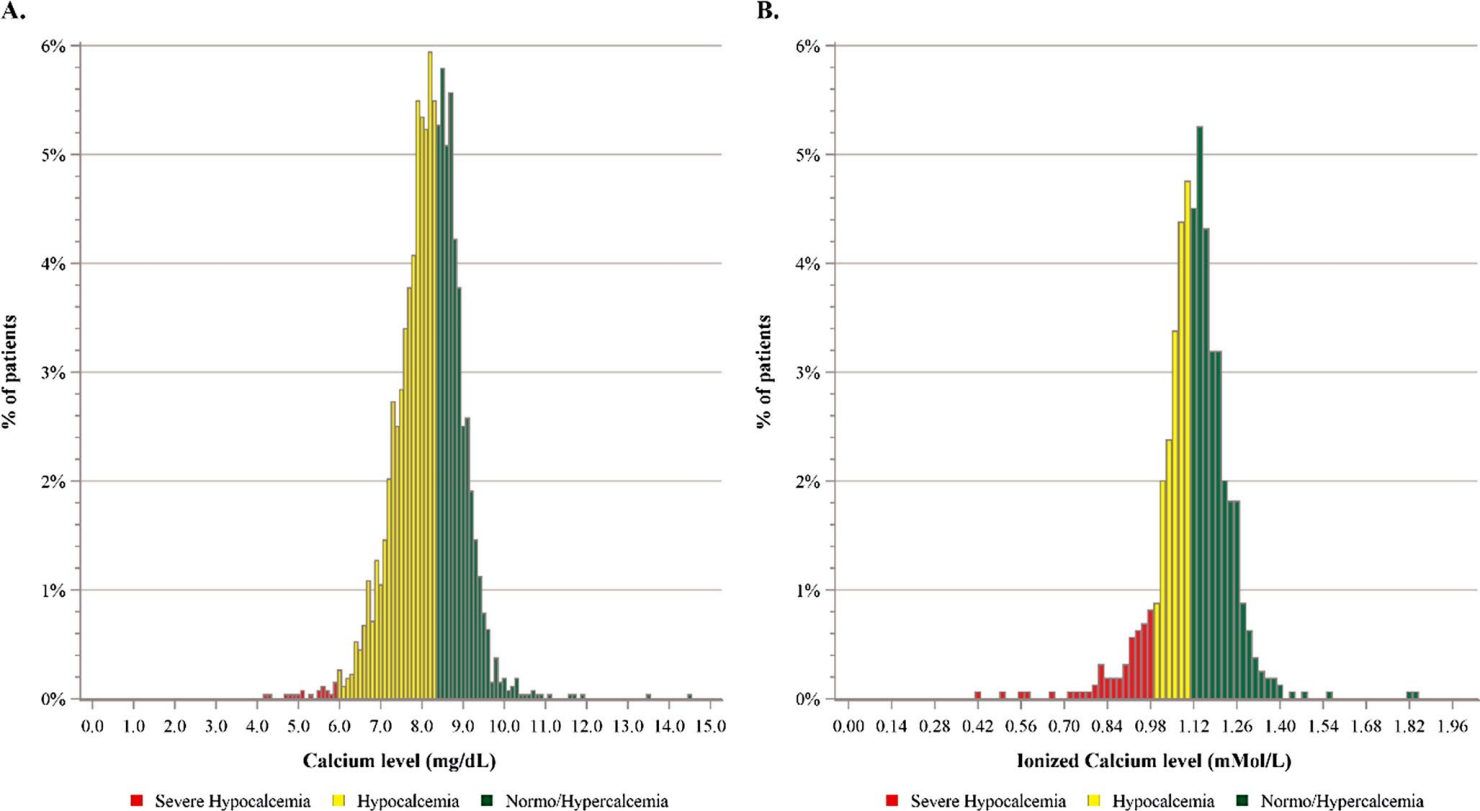
Calcium levels measured in the emergency department by severity of hypocalcemia

Of the 37,599 trauma patients evaluated between 2018 and 2024, 3,360 received 1–3 units of RBCs. A total of 8 were excluded due to missing calcium levels, and 672 were excluded due to length of stay < 4 days. The remaining 2,680 patients were stratified into four groups (normocalcemic versus hypocalcemic and received calcium supplementation versus did not receive calcium supplementation, Fig. 1). The distribution of patients presenting at various calcium levels is found in Fig. 2. The median time to administration was 35 min (IQR 15–108 min).

The four groups were significantly different in age (*p* < 0.001), sex (*p* < 0.001), and race (*p* = 0.040) (Table 1). These differences were driven by a lower proportion of males among the Normocalcemic, no-supplementation, a higher proportion of Black race in the hypocalcemic, no-supplementation group, and younger age among the hypocalcemic groups. Regarding injury characteristics, the four groups were significantly different in penetrating mechanism of injury, ISS, and SI (all *p* < 0.001), a result of lower injury severity for the normocalcemic, no-supplementation group. Patients who received supplementation were more likely to have a higher lactic acid among both normocalcemic and hypocalcemic patients.

Compared to normocalcemic, non-supplemented patients, there was a significantly increased two-fold odds of death among the hypocalcemic, supplemented group (OR 2.01, 95% CI 1.09–3.72) (Table 2); however, the interaction effects suggest a weak interactive effect (AP = 16.3%; ICR 0.32). Similarly, significantly increased associations for the hypocalcemic, supplemented group were observed for MI (OR 5.03, 95% CI 1.88–13.40) and sepsis (OR 5.44, 95% CI 1.17–25.37); however, the AP and ICR suggest weak interaction effects (MI: AP 5.8%, ICD 0.29; sepsis: AP 16.9%, ICD 0.90). That said, the hypocalcemic, supplemented group had significant effects and a noted interaction effect for AKI (OR 2.52, 95% CI 1.14–5.58; AP 40.0%; ICD 0.99), ARDS (OR 2.82, 95% CI 1.04–7.70; AP 57.3%; ICD 4.27), and PE (OR 4.26, 95% CI 1.67, 10.87; AP 30.9%); ICR 3.28).

In the supplemental propensity analysis, the observed associations for the hypocalcemic, supplemented group was observed among those with a lower propensity of calcium supplementation. In particular, significantly increased associations for this group was observed within the 10–14% propensity group for AKI (OR 5.52, 95% CI 1.69–17.98, Figure S1), ARDS (OR 8.74, 95% CI 2.29–33.39, Figure S2), DVT (OR 9.67, 95% CI 3.27–28.53, Figure S4), and PE (OR 4.51, 95% CI 1.17–17.36, Figure S8). Similar comparisons for cardiac arrest, mortality, infection, MI, sepsis, and stroke were not significant (Figures S3, S5-7, S9-10).

**Table 1:** Comparison of demographic and injury characteristics by serum calcium (Ca) level and calcium supplementation (SUP) status

	NormoCa/ No-SUP	NormoCa/ SUP	*p*-value*	HypoCa/ No-SUP	HypoCa/ SUP	*p*-value*	*p*-value†
*N*	820	118		1,488	254		
**DEMOGRAPHICS**							
Mean age, years (SD)	50.7 (21.2)	50.7 (20.0)	0.999	45.0 (19.3)	45.3 (19.7)	0.849	< 0.001
Male sex (%)	497 (60.6)	83 (70.3)	0.054	1,019 (68.5)	180 (70.9)	0.465	< 0.001
Race (%)							
White	523 (63.8)	75 (63.6)	0.978	870 (58.5)	164 (64.6)	0.042	0.040
Black	286 (34.5)	41 (35.3)		564 (37.9)	87 (34.3)		
Other	17 (2.1)	2 (1.7)		54 (3.6)	3 (1.2)		
**INJURY**							
Penetrating injury (%)	144 (17.6)	31 (26.3)	0.031	396 (26.6)	62 (24.4)	0.488	< 0.001
Mean ISS (SD)	16.5 (10.0)	20.6 (11.6)	< 0.001	20.1 (11.3)	21.6 (12.2)	0.062	< 0.001
ISS ≥ 16	393 (47.9)	77 (65.3)	0.001	916 (61.6)	166 (65.4)	0.263	< 0.001
Mean SI (SD)	0.83 (0.28)	0.97 (0.34)	< 0.001	0.89 (0.30)	1.08 (0.38)	< 0.001	< 0.001
SI > 1 (%)	186 (23.0)	46 (39.0)	< 0.001	438 (29.7)	130 (52.0)	< 0.001	< 0.001
AIS 3 + injury (%)							
Head	143 (17.4)	28 (23.7)	0.099	374 (25.1)	71 (28.0)	0.351	< 0.001
Thorax	235 (28.7)	54 (45.8)	< 0.001	551 (37.0)	128 (50.4)	< 0.001	< 0.001
Abdomen/Pelvis	122 (14.9)	26 (22.0)	0.058	368 (24.7)	70 (27.6)	0.348	< 0.001
Upper extremity	14 (1.7)	4 (3.4)	0.268	28 (1.9)	4 (1.6)	1.000	0.601
Lower extremity	341 (41.6)	48 (40.7)	0.921	568 (38.2)	97 (38.2)	1.000	0.419
**CLINICAL**							
Hemoglobin < 8 g/dL (%)	206 (25.2)	18 (15.3)	0.021	409 (27.5)	67 (26.4)	0.761	0.025
Lactic acid, mMol/L (%)							
≤2.2	275 (33.5)	16 (13.6)	< 0.001	344 (23.1)	41 (16.1)	< 0.001	< 0.001
2.3–3.9	280 (34.1)	40 (33.9)		499 (33.5)	67 (26.4)		
>3.9	265 (32.3)	62 (52.5)		645 (43.3)	146 (57.5)		
Mean Initial (SD)							
Calcium (mg/dL)	8.98 (0.50)	8.94 (0.49)	0.386	8.08 (0.73)	7.82 (0.85)	< 0.001	< 0.001
Ionized Calcium (mMol/L)	1.21 (0.09)	1.23 (0.11)	0.277	1.11 (0.12)	1.12 (0.12)	0.162	< 0.001
Mean Lowest (SD)							
Calcium (mg/dL)	8.87 (0.46)	8.84 (0.44)	0.481	7.83 (0.64)	7.61 (0.77)	< 0.001	< 0.001
Ionized Calcium (mMol/L)	1.20 (0.07)	1.20 (0.05)	0.806	1.09 (0.11)	1.09 (0.11)	0.638	< 0.001

* p-values for within-HypoCa groups estimated from chi-square or t-test for categorical and continuous variables, respectively

† p-values comparing all four groups estimates using Fisher’s exact test or analysis of variance for categorical and continuous variables, respectively

ISS: Injury Severity Score; SI: Shock Index; NormoCa: Normocalcemic; HypoCa: Hypocalcemic; SUP: received calcium supplementation; no-SUP: did not receive calcium supplementation

**Table 2:** Odds ratios (ORs) and associated 95% confidence intervals (CIs) for the association between serum calcium (Ca) and supplementation (SUP) status and selected clinical outcomes

	NormoCa/ No-SUP	NormoCa/ SUP	OR (95% CI)	HypoCa/ No-SUP	OR (95% CI)	HypoCa/ SUP	OR (95% CI)	AP	ICR
*N*	820	118		1,488		254			
Outcome n (%)									
AKI	17 (2.1)	3 (2.5)	1.38 (0.43–4.47)	29 (1.9)	1.11 (0.60–2.08)	12 (4.7)	2.52 (1.14–5.58)	40.0%	0.99
ARDS	9 (1.1)	0 (0.0)	0.38 (0.02–6.03)	10 (0.7)	0.58 (0.24–1.36)	6 (2.4)	2.82 (1.04–7.70)	57.3%	4.27
Cardiac arrest	7 (0.9)	3 (2.5)	3.74 (0.98–14.20)	23 (1.5)	1.98 (0.79–5.01)	4 (1.6)	2.45 (0.71–8.50)	7.7%	0.28
DVT	29 (3.5)	9 (7.6)	1.79 (0.83–3.88)	90 (6.0)	1.49 (0.96–2.30)	29 (11.4)	2.44 (1.40–4.26)	5.1%	0.12
PE	7 (0.9)	0 (0.0)	0.39 (0.02–6.59)	36 (2.4)	2.29 (1.05–5.01)	11 (4.3)	4.26 (1.67–10.87)	30.9%	3.28
Infection	27 (3.3)	3 (2.6)	0.89 (0.32–2.47)	74 (5.0)	1.30 (0.82–2.06)	11 (4.3)	1.04 (0.51–2.14)	-13.4%	-0.14
MI	9 (1.1)	4 (3.4)	4.38 (1.34–14.29)	14 (0.9)	1.34 (0.56–3.22)	9 (3.5)	5.03 (1.88–13.40)	5.8%	0.29
Sepsis	2 (0.2)	1 (0.8)	4.06 (0.60-27.38)	6 (0.4)	1.44 (0.37–5.67)	3 (1.2)	5.44 (1.17–25.37)	16.9%	0.90
Stroke	9 (1.1)	3 (2.5)	2.04 (0.59–7.03)	24 (1.6)	1.41 (0.67–2.98)	3 (1.2)	0.94 (0.27–3.24)	-	-1.49
Death	32 (3.9)	6 (5.1)	1.18 (0.49–2.86)	84 (5.7)	1.48 (0.96–2.27)	19 (7.7)	2.01 (1.09–3.72)	16.3%	0.32

* Estimated from a logistic regression utilizing Firth’s penalized likelihood and adjusted for age, sex, penetrating injury mechanism, Injury Severity Score ≥ 16, and shock index > 1; referent group is NormoCa/No-SUP

- AP for stroke not calculated due to the protective effect of the interactive effect

AKI: Acute Kidney Injury; ARDS: Acute Respiratory Distress Syndrome; DVT: Deep vein thrombosis; PE: Pulmonary embolism; Infection: surgical site infection, hospital-acquired pneumonia, ventilator-associated pneumonia, catheter-associated urinary tract infection, or central line-associated blood stream infection; MI: myocardial infarction; NormoCa: Normocalcemic; HypoCa: Hypocalcemic; SUP: received calcium supplementation; no-SUP: did not receive calcium supplementation

## Discussion

These data show that in trauma patients receiving 1–3 units of RBCs, those who were hypocalcemic and received early calcium supplementation were at increased odds of AKI, ARDS, DVT, sepsis, and MI. These findings suggest that there is a subset of patients meeting clinical triggers for transfusion in which early calcium administration is associated with harm. MTP protocols aim to improve outcomes by making resources rapidly available to patients with severe hemorrhage [15]; however, only 46% of patients initially predicted to require massive transfusion ultimately receive massive transfusion [16]. Routinely applying the same triggers for transfusion and empiric calcium supplementation may not be the ideal approach.

In this cohort of patients receiving 1–3 units of RBCs, only *n* = 165 (6.2%) developed severe hypocalcemia (< 1.0 mmol/L). This is consistent with other data showing severe hypocalcemia is most often associated with four or more units of combined blood product [17]. While there is a clear association between severe hypocalcemia and mortality in the literature [5, 6], the impact of mild-moderate hypocalcemia on outcomes is less clear [18]. Helsloot et al. found a U-shaped association between calcium and adverse outcomes, demonstrating an increase in coagulopathy and mortality above or below a range of 1.0-1.2 mmol/L [19]. This ideal range is slightly lower than a physiologic reference range, which suggests that a mild hypocalcemia is well-tolerated in this population. Much like TXA infused three hours after injury and early empiric cryoprecipitate increases death in certain subgroups [20, 21], it is reasonable to consider whether early, empiric calcium supplementation may have unintended consequences in this population.

Until further evidence is available, the authors recommend close monitoring of ionized calcium levels, reserving calcium supplementation for those with severe hypocalcemia (< 1.0 mmol/L) and the small group of trauma patients with ongoing massive transfusion requirements (> 3 units of RBCs). Even if MTP is activated, calcium supplementation should be carefully considered in patients that have a sustained and immediate response to small volume (1–3 units) of RBCs, even when mild to moderate hypocalcemia exists. However, the authors recognize that calcium metabolism is dynamic and may outpace the capability of point-of-care testing, especially among the sickest trauma patients. Therefore, the authors support early, empiric calcium administration during MTP in non- or transient responders with ongoing transfusion requirements. The ongoing (at the time of the current study) Calcium and Vasopressin following Injury Early Resuscitation (CAVALIER) trial (https://www.litesnetwork.org/cavalier) will evaluate empiric calcium before transfusion in a randomized, placebo-controlled fashion. Special attention should be paid to the subset of patients in this trial who ultimately require minimal transfusion.

Our study has several limitations. Because of the retrospective nature of this study and the limitations of the available data, we were unable to determine whether calcium was administered empirically or in a goal-directed fashion. We attempted a subgroup analysis comparing very early (< 1 h) to delayed, but the models failed to converge. Therefore, future research should investigate whether the timing of calcium administration modifies the observed associations with adverse clinical events. Calcium dosing was also not available, and future work should address dose-response relationships. We were also unable to stratify patients by severity of hypocalcemia–few (*n* = 165, 6.2%) patients developed severe hypocalcemia–and by the specific number of red blood cell units transfused due to issues with the maximum likelihood models not converging for the outcomes with a low prevalence; future research should address this limitation to examine whether the observed associations vary by severity of hypocalcemia or number of RBCs transfused. Additionally, the observed associations among the hypocalcemic, supplementation group may be due in part to being a marker of disease severity; however, this concern is mitigated by the finding in the supplemental propensity analysis that associations were stronger among those with a low predicted probability of calcium supplementation. Patients were excluded if their length of stay was less than 4 days since this is a typical timeframe to develop the complications of interest. However, this may introduce some selection bias by removing both early deaths and less severely injured patients. Finally, the observed associations may be biased by residual confounding as the analytical dataset did not contain information on variables such as pre-hospital blood transfusion and calcium supplementation, time to hemorrhage control, crystalloid use, and comorbidities. Due to these limitations, it must be noted that the current associations are, at this point, correlations and causation cannot be assumed.

## Conclusions

The current study suggests that the use of calcium supplementation is associated with adverse outcomes—particularly AKI, ARDS, and PE—in the absence of a beneficial effect on mortality. As a result, the use of early, empiric calcium supplementation in conjunction with early, minimal transfusion should be carefully considered.

## Supplementary Information

Below is the link to the electronic supplementary material.

Supplementary Material 1

## Data Availability

The dataset used in this study is protected for patient privacy. Requests for access made to the corresponding author will be considered on a case-by-case basis.
