# Supplementary material for: Early calcium replacement after minimal blood transfusion is associated with adverse outcomes
Source: Eur J Trauma Emerg Surg. 2026 Aug 7;52(1):238. doi: 10.1007/s00068-026-03282-6 (PMC13451501; doi:10.1007/s00068-026-03282-6)
Supplement: Supplementary file 1 [file 68_2026_3282_MOESM1_ESM.docx]

# Supplemental Figures

**Figure S1.** Odds ratios (ORs) and 95% confidence intervals for the association between calcium status (hypocalcemic [HypoCa] and normocalcemic [NormoCa]) and calcium supplementation (SUP) status and acute kidney injury (AKI) stratified by calcium supplementation propensity score.

**Figure S2.** Odds ratios (ORs) and 95% confidence intervals for the association between calcium status (hypocalcemic [HypoCa] and normocalcemic [NormoCa]) and calcium supplementation (SUP) status and acute respiratory distress syndrome (ARDS) stratified by calcium supplementation propensity score.

**Figure S3.** Odds ratios (ORs) and 95% confidence intervals for the association between calcium status (hypocalcemic [HypoCa] and normocalcemic [NormoCa]) and calcium supplementation (SUP) status and cardiac arrest stratified by calcium supplementation propensity score.

**Figure S4.** Odds ratios (ORs) and 95% confidence intervals for the association between calcium status (hypocalcemic [HypoCa] and normocalcemic [NormoCa]) and calcium supplementation (SUP) status and deep vein thrombosis (DVT) stratified by calcium supplementation propensity score.

**Figure S5.** Odds ratios (ORs) and 95% confidence intervals for the association between calcium status (hypocalcemic [HypoCa] and normocalcemic [NormoCa]) and calcium supplementation (SUP) status and death stratified by calcium supplementation propensity score.

**Figure S6.** Odds ratios (ORs) and 95% confidence intervals for the association between calcium status (hypocalcemic [HypoCa] and normocalcemic [NormoCa]) and calcium supplementation (SUP) status and infection stratified by calcium supplementation propensity score.

**Figure S7.** Odds ratios (ORs) and 95% confidence intervals for the association between calcium status (hypocalcemic [HypoCa] and normocalcemic [NormoCa]) and calcium supplementation (SUP) status and myocardial infarction (MI) stratified by calcium supplementation propensity score.

**Figure S8.** Odds ratios (ORs) and 95% confidence intervals for the association between calcium status (hypocalcemic [HypoCa] and normocalcemic [NormoCa]) and calcium supplementation (SUP) status and symptomatic pulmonary embolism (PE) stratified by calcium supplementation propensity score.

**Figure S9.** Odds ratios (ORs) and 95% confidence intervals for the association between calcium status (hypocalcemic [HypoCa] and normocalcemic [NormoCa]) and calcium supplementation (SUP) status and sepsis stratified by calcium supplementation propensity score.

**Figure S10.** Odds ratios (ORs) and 95% confidence intervals for the association between calcium status (hypocalcemic [HypoCa] and normocalcemic [NormoCa]) and calcium supplementation (SUP) status and stroke stratified by calcium supplementation propensity score.
